# Mobile Robot Positioning with 433-MHz Wireless Motes with Varying Transmission Powers and a Particle Filter

**DOI:** 10.3390/s150510194

**Published:** 2015-04-30

**Authors:** Adrian Canedo-Rodriguez, Jose Manuel Rodriguez, Victor Alvarez-Santos, Roberto Iglesias, Carlos V. Regueiro

**Affiliations:** 1 CITIUS, University of Santiago de Compostela, R/ Jenaro de la Fuente Dominguez s/n, 15782 Santiago de Compostela, Spain; E-Mails: roberto.iglesias.rodriguez@usc.es (R.I.);; 2 Situm Technologies S. L. R/ de Lope Gomez de Marzoa s/n. Edif. Feuga Desp. 16. 17505 Santiago de Compostela, Spain; E-Mail: victor@situm.es; 3 Electronics Department, University of Alcala, Carretera Madrid-Barcelona Km 33.600, 28871 Alcala de Henares (Madrid), Spain; E-Mail: jmra@depeca.uah.es; 4 Department of Electronics and Systems, University of A Coruña, Campus de Elviña s/n, 15071 A Coruña, Spain; E-Mail: cvazquez@udc.es (C.V.R.)

**Keywords:** wireless localization, WiFi localization, motes, particle filters, robot localization

## Abstract

In wireless positioning systems, the transmitter's power is usually fixed. In this paper, we explore the use of varying transmission powers to increase the performance of a wireless localization system. To this extent, we have designed a robot positioning system based on wireless motes. Our motes use an inexpensive, low-power sub-1-GHz system-on-chip (CC1110) working in the 433-MHz ISM band. Our localization algorithm is based on a particle filter and infers the robot position by: (1) comparing the power received with the expected one; and (2) integrating the robot displacement. We demonstrate that the use of transmitters that vary their transmission power over time improves the performance of the wireless positioning system significantly, with respect to a system that uses fixed power transmitters. This opens the door for applications where the robot can localize itself actively by requesting the transmitters to change their power in real time.

## 1. Introduction

Mobile robot localization is the problem of determining the pose (position and orientation) of a robot relative to a map. This problem is one of the most important in mobile robotics, because most robotic tasks require knowledge of the robot pose [1]. Sonar sensors [2], cameras [3] and, above all, 2D laser range finders [1] mounted on the robot have been three popular choices to locate a robot indoors. Another alternative is to infer the robot position based on the characteristics of the signal received from wireless transmitters placed in the environment (wireless localization). There are a number of technologies that have been used for wireless localization [4–6], such as RFID, WiFi, Bluetooth, ZigBee, GSM or UWB.

Nevertheless, each sensor has limitations, and no sensor is applicable to all situations [7]. For instance, the 2D laser provides information that can lead to very accurate (sub-meter) localization estimates. However, 2D laser range finders suffer from several well-known issues [7,8]: perception limitations (e.g., they cannot detect glass walls), occlusion in crowded environments (e.g., when operating in a museum with people around the robot), inability to distinguish among similar areas (e.g., corridors), failures when dealing with changes in the environment, *etc*. Most of these issues are also common when using cameras [3,9]. On the other hand, other alternatives, such as wireless localization, cannot provide such a level of accuracy, but they are robust against the situations that we have mentioned before. For these reasons, in the past, we have solved the problem of mobile robot localization by fusing the information of a 2D laser rangefinder, a WiFi card and a magnetic compass [8]. The key idea is to increase the robustness and redundancy of localization systems by combining the information of sensors of different natures, which will fail in different situations. We have applied these techniques to a tour-guide robot that operates in challenging crowded environments [10], and the fusion of different sensors has proven to result in a very robust positioning algorithm.

Among the range of wireless positioning technologies available, WiFi positioning is the most popular. WiFi positioning aims to estimate the robot position from the signal power received from different WiFi access points (e.g., a high signal power level indicates that the robot is close to the corresponding AP). These transmitters are mostly commercial; therefore, they use similar transmission powers that cannot be modified. We consider that the use of transmitters that can vary their transmission power could lead to a number of benefits. On the one hand, each environment is different, and using a default transmission power may not be the most optimal solution. On the other, different parts of the environment or different situations might require different levels of accuracy and robustness and, therefore, different transmission powers. For instance, the signal attenuation of a high-power transmitter might not be noticeable in a small room, so it might not be possible to distinguish where the robot is within the room. On the contrary, a low-power transmitter might not be received outside a region close to it, so we could know whether the robot is within this region. Even more, different transmission powers lead to different signal propagations, which provide different information. Therefore, the use of transmitters with varying transmission power will increase the discrimination ability of the localization system. In the extreme, these transmission powers could be changed by the robot itself. This would allow the robot to discard localization hypotheses actively depending on different criteria: the quality of the current localization estimate, the task at hand, the part of the environment where the robot is, *etc*.

Taking this into account, in this paper, we will explore the use of wireless transmitters that are able to vary their transmission power. To this extent, we will use specifically designed wireless sensor nodes [11,12], also known as motes. We could have used other alternatives, such as commercial WiFi APs or Bluetooth beacons, but motes are a flexible and convenient solution for our purposes. Moreover, motes have several characteristics that make them very appealing for mobile robot applications:

- Control over the software and hardware of the mote. This enables the adjustment of the transmission power, among other properties.
- Communication capabilities. Motes can be used for communication among robots and environment elements.
- Low power consumption. Motes usually consume much less power that their WiFi APs. What is more important, designers have full control over it.
- Homogeneous hardware. It is known that different WiFi receptors have different reception properties, which constitute a challenge for wireless localization algorithms [13]. Having homogeneous hardware limits this phenomenon and simplifies the working conditions of the robots, which enhances the robustness and reduces the cost of design and deployment.
- Several frequency bands. This might be interesting if we want to avoid interferences with existing systems (such as WiFi networks) or to comply with the radiation policies of the application environment (e.g., in hospitals or industrial plants).

We have designed motes that operate in the 433-MHz band. These motes can be produced for less than 15€ each, allow the modification of several transmission parameters and have a very small power consumption (average current lower than 0.1 mA^@^ 3 VDCin real operation). In a typical setting, several motes are deployed in the environment, and one mote is placed on board each robot. The motes placed in the environment transmit information packets periodically, and the mote on board the robot measures the signal power received and estimates the robot position.

In our system, to estimate the robot position, we will use a particle filter [1], a state-of-the-art algorithm typically used in mobile robot localization. This method maintains a probabilistic estimate of the robot position, which evolves based on the robot movement and the sensor observations. This has shown to be superior over methods that estimate a new robot position when new data arrives, without taking into account previous estimates [14]. Other probabilistic algorithms, such as Kalman filters and their most popular variants [1], were considered, as well. However, they represent the state (pose of the robot in our case) using a Gaussian random variable, while particle filters can deal with arbitrary probabilistic representations. This allows us to maintain several localization estimates simultaneously, which is very useful when sensor readings are not sufficiently discriminative to estimate a unique position. Moreover, with particle filters, the probabilistic mapping between sensor measurements and position candidates can follow any probabilistic distribution. This provides us with great flexibility when modeling the localization sensors.

We would like to point out that our objective is not to compare motes against WiFi localization, nor to suggest a new positioning algorithm. Instead, our objective and main contribution is to test whether the use of varying transmission powers increases the quality of a robot positioning system. This is an issue that has not been rigorously addressed in the past. First of all, we compare mote-based localization and WiFi-based localization using three different commercial WiFi receivers. This analysis shows that, when using fixed power transmitters, there are no differences among the performances of both systems. Then, we perform an experimental comparison among different fixed transmission powers for wireless localization. With this analysis, we demonstrate that different transmission powers lead to different performances, which indicates the importance of this parameter. Finally, we perform an experimental analysis where our motes change their transmission power periodically. With this analysis, we show that the use of varying transmission powers tends to increase the performance of wireless positioning systems.

The rest of the paper is organized as follows. Section 2 contains a review of the related work, including other positioning systems, techniques and algorithms. Section 3 describes the hardware and the software of the motes. Section 4 describes the localization algorithm based on particle filters. Section 5 describes the observation model proposed for the motes and the methodology followed to capture the calibration data and to calibrate this model. Finally, Section 6 contains the experimental results.

## 2. Related Work

Most of the positioning works with motes have been devoted to the localization of the static motes that form the sensor network [15,16]. There have been a few attempts to use motes in person [17] and robot positioning applications [18,19], but the examples are scarce. However, motes are closely related to other wireless technologies used in indoor positioning [4–6], such as systems based on WiFi, RFID, UWB, Bluetooth, TV or GSM. Therefore, knowing the techniques that have been applied with these technologies can be useful to construct an indoor positioning system based on motes.

Among the wireless positioning alternatives, WiFi positioning has been the most popular alternative by far. WiFi localization is usually based on fingerprinting [14,20]. Fingerprinting refers to techniques that: (1) at the calibration stage, collect features or fingerprints of the wireless signal and the location where they were measured to build a radio map; and (2) at the use stage, estimate the position of the receptor by matching online measurements with the radio map. Usually, RSSI (received signal strength indication) features are used, which are related to the signal power received from the access points.

There are two basic kinds of radio maps that can be constructed with fingerprinting techniques: model-based and empirical maps [20]. Model-based maps are defined by a set of parameters that specify the characteristics of the environment (e.g., walls, materials, *etc.*) and/or the characteristics of the signal propagation. These parameters are usually adjusted using calibration data. On the other hand, empirical methods (which tend to achieve better results [21,22]), work directly with the fingerprints to build radio maps. There are two kinds of empirical maps: deterministic and probabilistic maps [20]. Deterministic maps assign a single value to each position of the map, such as a fingerprint in that position, or an average of the closest fingerprints. The most important drawback of these maps is that a single value cannot capture the random nature of wireless signals. As a solution, probabilistic maps characterize the wireless signal at each position using probability distributions [14] (e.g., Gaussian, log-normal, Weibull, *etc.*). In this paper, we will construct probabilistic maps using the Gaussian process regression technique [23], which is able to estimate the average and typical deviation RSSI values at every map position.

On the other hand, almost any algorithm from the fields of machine learning and estimation could be used as a position estimation method. Usually, estimation methods are divided into two groups [14,20]: deterministic and probabilistic. Deterministic methods estimate the location of the receptor directly from the value of the measurements received. Techniques, such as artificial neural networks [24,25], support vector machines [8,26] and nearest neighbors and their variants [14,21,27], have been used to implement deterministic localization techniques. On the other hand, probabilistic methods estimate the position of the device as part of a random process. Usually, they integrate the measurements sequentially and exploit information about the movement of the device or about the topology of the environment. It has been shown that probabilistic techniques tend to have better results than deterministic ones [14]. Probabilistic methods are usually based on Bayesian inference [14], hidden Markov models [28] or particle filters [8,29].

Probabilistic techniques maintain a probabilistic model of the state of a system (e.g., robot), which evolves over time and is periodically observed by a sensor (or sensors). From all of the probabilistic estimation algorithms, Bayesian filtering approaches, such as Kalman filters and particle filters, are by far the most popular. Kalman filters work well when [1]: (1) the robot motion is linear; (2) the motion and sensor noises are white, Gaussian and can be modeled accurately; (3) there is an explicit and unimodal mapping (observation model) between states and observations; and (4) the best estimate of the state is unique (unimodal probability distribution over the state space). Some of these conditions do not hold in the context of robot localization. For instance, the robot movement is usually non-linear, and in practice, it may be hard to model the observation models and their noises explicitly using linear Gaussian models. Above all, observation models and, therefore, state probability distributions are rarely unimodal.

Particle filters are a powerful, yet efficient alternative to Kalman filters. Particle filters do not make any of the previous assumptions: they work with non-linear non-Gaussian systems with multi-modal probability distributions, where there is no explicit mapping between sensor observations and system states. Moreover, particle filters are very robust, even if the system and sensor noises are poorly estimated. For these reasons, particle filters have been a popular approach to solve the problem of robot localization [1].

## 3. Motes Description

**Figure 1:**
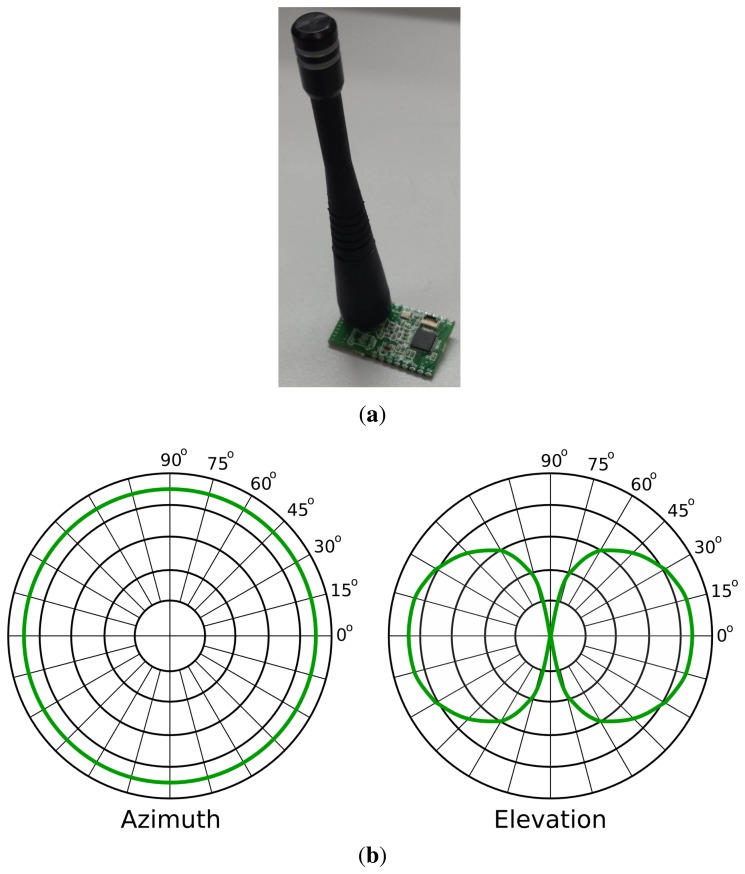
(**a**) Prototype of a mote using a CC1110 sub-1-GHz SoC with a monopole antenna; (**b**) radiation pattern of the monopole antenna for an arbitrary transmission power.

We have developed a prototype of a mote network using a CC1110 SoC (Figure 1a). These CC1110 combine an industry standard-enhanced 8051 MCU and an excellent performance RF transceiver CC1101. Our motes operate at 3 V and consume less than 10 uA in sleep mode and 34 mA when transmitting at 10 dBm (maximum power). Therefore, they consume approximately 0.1 W in the worst case. Just to give a comparison, we have measured that the power consumption of a commercial router (Linksys WAG200G) is 4.5 W, even when it is not transmitting information. Every mote is powered by two AAA batteries with an estimated battery life of several months (assuming a 2-ms data burst every 1 s at maximum transmission power, 10 dBm). We have programmed the motes to operate at a 10-kbps data rate, using GFSKmodulation in the 433-MHz ISM band with 19 kHz of deviation and 100 kHz of RXbandwidth filters.

We have equipped each mote with a 1.8-dBimonopole antenna from MaxStream (Figure 1a). The radiation pattern of this antenna is illustrated in Figure 1b. In the azimuth radiation graph, we observe that the antenna radiates equally in all directions of the plane parallel to the ground. We will estimate the position of the robot in this plane (2D robot localization). In the elevation radiation graph, we observe that the antenna also radiates in directions that are not parallel to the ground. Therefore, in principle, the motes could be used to estimate the attitude of a receiver. For instance, they could distinguish among different floors or the attitude of a drone, although this goes beyond the scope of this paper. Moreover, in this last case, other sensors (e.g., sonar sensors) could provide higher precision, because indoor ceilings are rarely high, and the attenuation from the ground to the ceiling would not be perceivable.

Our motes can be used both for communication and localization purposes, but in this paper, we will focus on the latter. In this regard, we have programmed some of the motes to send data packages periodically (transmitter motes). The data sent in the packages indicate the output power at which the packages are transmitted and the ID of the transmitting mote. On the other hand, the receiver motes were programmed to receive these packages and to measure the power of the signal received. These motes are placed on board the robot.

## 4. Localization Algorithm

In this section, we will present the particle filter algorithm, a well-known localization algorithm that can be used both for localization with WiFi and motes. In essence, this algorithm aims to estimate, at every instant *t*, the pose of the robot (state) *s_t_* = (*x_t_*, *y_t_*, *θ_t_*) with respect to a map. Here, (*x_t_*, *y_t_*) represents the position in Cartesian coordinates and *θ_t_* the orientation. This is done based on: (1) perceptual information *z_t_*; and (2) control data *u_t_*. In our case, *z_t_* represents the signal power received by the robot from the motes placed in the environment and *u_t_* the robot movement as provided by odometry encoders. In addition, we will estimate Σ*_t_*(*s*) (the covariance of *s_t_*). In order to accomplish our goal, our system iterates over a two-step process:

1. Pose probability estimation (Sections 4.1 and 4.2): This step computes the pose probability distribution over all possible robot poses. This distribution is usually called the belief distribution, and it represents the belief that any possible pose refers to the actual current position and orientation of the robot. As the pose of the robot changes over time, so does the belief distribution *bel*(*s_t_*).
2. Pose estimation (Section 4.3): Estimation of the most likely current pose *st* from the pose probability distribution *bel*(*s_t_*).

### 4.1. Recursive Bayes Filtering to Estimate the Pose Probability Distribution

We will use the Bayesian filtering approach to estimate the pose of the robot. Under this approach, the belief distribution is the posterior probability density function of the pose based on all of the available information. This information consists of: (1) the set of actions taken by the robot *u_t_*_:1_ = {*u_t_*, *u*_*t*−1_, …, *u*_1_}; and (2) the set of received sensor measurements *z_t_*_:1_ = {*z_t_*, *z_t_*_−_*_1_*, …, *z*_1_} [1,30].

(1) $$\text{bel}\left(s_{t}\right)=p\left(s_{t}|z_{t:1},u_{t:1}\right)$$

Equation (1) requires storing all of the information received and processing it as a batch when new data becomes available. Instead, a recursive filter is a much more convenient solution, since it allows processing the received data sequentially and discarding it after that. This filter can be derived using the Bayes rule [1]:

(2) $$\text{bel}\left(s_{t}\right)\propto p\left(z_{t}|s_{t},z_{t-1:1},u_{t:1}\right)p\left(s_{t}|z_{t-1:1},u_{t:1}\right)$$

and the law of total probability [1]:

(3) $$\text{bel}\left(s_{t}\right)\propto p\left(z_{t}|s_{t},z_{t-1:1},u_{t:1}\right)\int p\left(s_{t}|s_{t-1},z_{t-1:1},u_{t:1}\right)\text{bel}\left(s_{t-1}\right)ds_{t-1}$$

Furthermore, it is common to use the Markov assumption [1], which states that past and future data are independent if one knows the current state (pose). Therefore, provided the current state, past states or data are not relevant to future predictions. In this case, the knowledge of *s_t_*_−1_ and *u_t_* suffices to predict *s_t_*:

(4) $$p\left(s_{t}|s_{t-1},z_{t-1:1},u_{t:1}\right)=p\left(s_{t}|s_{t-1},u_{t}\right)$$

Similarly, the knowledge of *s_t_* is enough to predict *z_t_*:

(5) $$p\left(z_{t}|s_{t},z_{t-1:1},u_{t:1}\right)=p\left(z_{t}|s_{t}\right)$$

Therefore, Equation (3) can be rewritten as:

(6) $$\text{bel}\left(s_{t}\right)\propto p\left(z_{t}|s_{t}\right)\int p\left(s_{t}|s_{t-1},u_{t}\right)\text{bel}\left(s_{t-1}\right)ds_{t-1}$$

**Figure 2:**
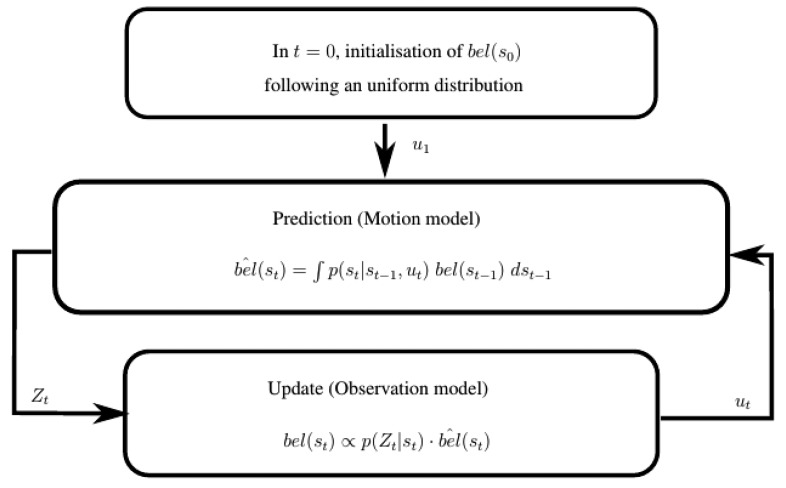
Block diagram of the recursive Bayes filter for mobile robot localization.

This is the general form of the recursive Bayes filter, represented in Figure 2. The first belief distribution *bel*(*s*_0_) can be initialized randomly. Then, the filter performs iteratively in two stages: prediction and update.

#### 4.1.1. Prediction

This stage predicts a new belief distribution *bêl*(*s_t_*) of the pose of the robot from *bel*(*s_t_*_−1_), considering the current robot movement *u_t_*:

(7) $$b\hat{e}l\left(s_{t}\right)=\int p\left(s_{t}|s_{t-1},u_{t}\right)\text{bel}\left(s_{t-1}\right)ds_{t-1}$$

The term *p*(*s_t_*|*s_t_*_−1_, *u_t_*) is called the motion model of the robot. It describes, from a probabilistic perspective, the evolution of the pose when only the actions taken by the robot are considered. This model depends on the specific kinematics of the robot (*i.e.*, how much its position will evolve considering the angular and linear velocities performed).

#### 4.1.2. Update

The update stage uses the latest sensor measurements *z_t_* to correct the belief distribution previously predicted *bêl*(*s_t_*), producing the true posterior distribution *bel*(*s_t_*):

(8) $$\text{bel}\left(s_{t}\right)\propto p\left(z_{t}|s_{t}\right)\cdot b\hat{e}l\left(s_{t}\right)$$

**Figure 3:**
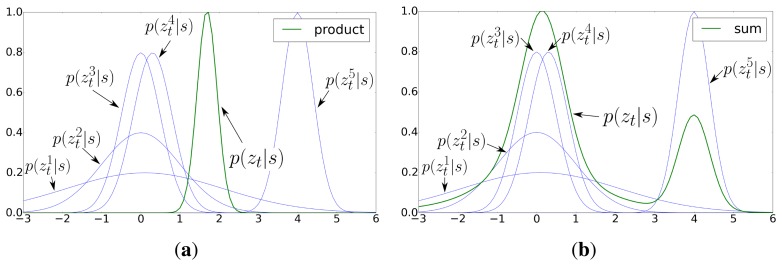
Likelihood distribution resultant from: (**a**) Equation (9) (product); (**b**) Equation (10) (sum). Each blue line represents an individual function **(INLINE)** and the green line the fusion of these functions using Equation (9) or (10), respectively.

The function *p*(*z_t_*|*s_t_*) is called the observation model: it specifies the probability of receiving a certain measurement *z_t_* provided that the robot is in a certain pose *s_t_*. In our case, it represents the probability of receiving a certain vector of signal powers, when the robot is at pose *s_t_*. This vector of signal power is $z_{t}=\left\{z_{t}^{1},\ldots ,z_{t}^{n_{t}^{z}}\right\}$, where $n_{t}^{z}$ is the number of transmitters available at time *t* and $z_{t}^{i}$ is the power in dBms of the *i*-th wireless transmitter at time *t*. Therefore, *p*(*z_t_*|*s_t_*) is a joint probability function, which is hard to estimate in practice (especially when the number of transmitters is high). Instead, we can assume that the transmitters are conditionally independent given an arbitrary pose *s*:

(9) $$p\left(z_{t}|s\right)=\prod_{k=1}^{n_{t}^{z}}p\left(z_{t}^{k}|s\right)$$

where $p\left(z_{t}^{k}|s\right)$represents the probability that the robot receives a signal power $z_{t}^{k}$ from the *k*-th transmitter, assuming that the robot is at pose *s*. In our experience, this approximation is not robust, because it depends too much on the output of each individual function $p\left(z_{t}^{k}|s\right)$. This is because the combination is a product of probabilities, so even if most functions agree on a certain pose, a single function that has a value near zero around that pose will cause the product to fall close to zero. This is represented in Figure 3, where even if most models $p\left(z_{t}^{k}|s\right)$ would predict a position around *s* ≈ 0 (probably correctly), a single model ( $p\left(z_{t}^{5}|s\right)$) is enough to distort the prediction *p*(*z_t_*|*s*) so that the maximum falls at the intersection between all of the distributions (probably incorrect). This represents an issue, because even small noises on the power received from the motes can have a big impact on the robustness of the predictions. In our experience, a simple voting scheme can be a much more robust solution:

(10) $$p\left(z_{t}|s\right)=\sum_{k=1}^{n_{t}^{z}}p\left(z_{t}^{k}|s\right)$$

The underlying idea is that each individual model can contribute to the final prediction, but no model should have such an influence as in Equation (9). Essentially, Equation (10) solves this problem by aggregating the individual probabilities. This is represented in Figure 3, where the maximum value of *p*(*z_t_*|*s*) is achieved where most $p\left(z_{t}^{k}|s\right)$ achieve their respective maximum (where most distributions agree), which is probably the correct prediction. The resultant distribution is therefore less affected by individual fluctuations. Taking this into account, we can rewrite Equation (8) as:

(11) $$\text{bel}\left(s_{t}\right)\propto b\hat{e}l\left(s_{t}\right)\sum_{k=1}^{n_{t}^{z}}p\left(z_{t}^{k}|s_{t}\right)$$

Equation (8) corrects *bêl*(*s_t_*) based on the similarity between *z_t_* (power received from the transmitters at time *t*) and the power expected to be received at each pose *s_t_*. Under this approach, the most probable poses will be those with the highest similarity between expected and received signal power. We will explain in Section 5 how we obtain the observation model of each transmitter.

### 4.2. Implementation of the Recursive Bayes Filtering with Particle Filters

It is not straightforward to compute *bel*(*s_t_*); therefore, approximations, such as the sequential importance sampling (SIS) algorithm (also known as particle filter) [1,30–32], are used in practice. This algorithm implements the recursive Bayesian filter described above using sequential Monte Carlo simulations. This method assumes that *bel*(*s_t_*) can be represented by a set of *n^p^* samples, usually called particles. The sample set will be represented as $P_{t}=\left\{\begin{array}{cc} P_{t}^{i}=\left(s_{t}^{i},\omega_{t}^{i}\right), & \forall i\in \left\{1,\ldots ,n^{p}\right\} \end{array}\right\}$, where $s_{t}^{i}$ is the pose of the sample and $\omega_{t}^{i}$ a weight associated to the sample (the sum of the weights must add up to one). Taking this into account, the *bel*(*s_t_*) function will be represented as:

(12) $$\text{bel}\left(s_{t}\right)\approx \sum_{i=1}^{n^{p}}\omega_{t}^{i}\cdot \delta \left(s_{t}^{i}-s_{t}\right)$$

where $\delta \left(s_{t}^{i}-s_{t}\right)$ is Dirac's delta function centered at $s_{t}^{i}$. Note that the more weight the particle has, the more likely its pose is.

The particle filter performs as follows. First of all, a set of particles *P*_0_ is generated with random poses $s_{0}^{i}$ and equal weights $\omega_{0}^{i}$. Then, the algorithm iterates over the following steps:

1. Prediction: Every time the robot moves (*u_t_*), the algorithm samples a new pose $s_{t}^{i}$ for each particle taking into account the robot motion model:
  (13) $$\begin{array}{cc} s_{t}^{i}∼p\left(s_{t}|s_{t-1}^{i},u_{t}\right) & \forall i\in \left\{1,\ldots ,n^{p}\right\} \end{array}$$
  This can be seen as a displacement *u_t_* of each particle. To accommodate the error of the odometry of the robot, we add some random noise in position and orientation [1].
2. Update: Then, the weight of each particle is updated taking into account the latest measurements *z_t_*:
  (14) $$\omega_{t}^{i}\propto \omega_{t-1}^{i}p\left(z_{t}|s_{t}^{i}\right)=\omega_{t-1}^{i}\sum_{k=1}^{n_{t}^{z}}p\left(z_{t}^{k}|s_{t}^{i}\right)$$

Note that each particle integrates the past sensor information (using the previous weight) and the fusion of the current sensor information. After the update step, the set of weights is normalized.

#### 4.2.1. Resampling

After a while, all of the particles, except one, will have negligible weights. This is known as the sample depletion phenomenon [32,33]. The degree of depletion can be defined as the effective number of effective particles [32,33]:

(15) $$N_{\text{eff}}=\frac{1}{\sum_{i=1}^{n^{p}}{\left(\omega_{t}^{i}\right)}^{2}}$$

When all of the weights are equal, we have the maximum number of effective particles (*N_eff_* = *n^p^*), and therefore, the lowest degree of depletion. Conversely, when only one particle accumulates all of the weight, we have the lowest number of effective particles (*N_eff_* = 1) and the highest degree of depletion.

The depletion phenomenon can be corrected by performing a resampling step when the number of effective particles falls below a certain threshold value (e.g., $\frac{2}{3}n^{p}$). This step consists of the construction of a new set of particles from the current one. First, we take *n^nr^* particles from the current set with probability proportional to their weight using the low variance resampling technique [1] (we may repeat particles). Then, we generate a variable number *n^r^* of random particles, such that *n^nr^* + *n^r^* = *n^p^*.

The generation of random particles allows the algorithm to recover when it converges erroneously to a wrong pose. In order to compute *n^r^*, we keep a long-term average $\omega_{t}^{l}$ and a short-term average $\omega_{t}^{s}$ of the weight of the particle set:

(16) $$\omega_{t}^{l}=\omega_{t-1}^{l}+\alpha^{l}\cdot \left(\frac{1}{n^{p}}\sum_{i=1}^{n^{p}}\omega_{t}^{i}-\omega_{t-1}^{l}\right)$$

(17) $$\omega_{t}^{s}=\omega_{t-1}^{s}+\alpha^{s}\cdot \left(\frac{1}{n^{p}}\sum_{i=1}^{n^{p}}\omega_{t}^{i}-\omega_{t-1}^{s}\right)$$

(18) $$n^{r}=\left\lceil n^{p}\cdot \max\left(0,1-\frac{\omega_{t}^{s}}{\omega_{t}^{l}}\right)\right\rceil$$

where 0 < *α^l^* << *α^s^* < 1. The ratio $\omega_{t}^{s}/\omega_{t}^{l}$ estimates whether the quality of the particle set is increasing (increasing ratio) or decreasing (decreasing ratio). Therefore, the more this ratio decreases, the more random particles we generate.

### 4.3. Pose Estimation

At this point, we have computed *bel*(*s_t_*), and we need to estimate the most likely pose *s_t_*:

(19) $$s_{t}=\underset{s}{\text{argmax}}\left\{\text{bel}\left(s_{t}\right)\right\}$$

Since we are using the particle filtering approach, we have to estimate this pose from the particle set *P_t_*. A popular approach is to estimate this pose as the weighted mean over all the particles [1]. However, in practice, this might not give good results (e.g., when we have two or more bulks of particles in different positions, it will estimate a position between the sets).

To solve these issues, we propose to use a clustering-based process. Since most particles will concentrate in a few regions (the most likely regions), we should be able to detect clusters of particles (pose hypotheses) and select one of them. To this extent, we perform the following two-step process:

1. Hypothesis generation: First of all, we use agglomerate clustering [34] to group the particles into clusters. Each particle will be assigned to its closest cluster, provided that the distance between the particle and the cluster centroid is lower than a threshold distance in position *dist*_*th*−*xy*_ and in orientation *dist*_*th*−*θ*_. Otherwise, the particle will create a new cluster. Each cluster *i* can be interpreted as a hypothesis about the position of the robot. We will represent each hypothesis as $H_{t}^{i}=\left\{\Omega_{t}^{i},\mu_{t}^{i},\sum_{t}^{i}\right\}$, where $\Omega_{t}^{i}$ is the total weight of the particles contained in the cluster *i*, $\mu_{t}^{i}$ is the average pose of the particles of the cluster and $\sum_{t}^{i}$ is their covariance matrix. The influence of each particle on these two statistics (mean and covariance) is proportional to its weight.
2. Hypothesis selection: We will select the hypothesis that accumulates more weight, provided that this accumulated weight exceeds a certain threshold: $\Omega_{t}^{i}>\Omega_{th}\left(\Omega_{th}\in \left[0.5,1\right]\right)$. Then, the robot pose *st* will be $\mu_{t}^{i}$, and the covariance Σ*_t_*(s) will be $\sum_{t}^{i}$. In the next step, this hypothesis will be chosen again if $\Omega_{t}^{i}>1-\Omega_{th}$. This increases the stability of the hypothesis selection.

## 5. Observation Model for Wireless Localization

We have explained that the observation model *p*(*z_t_*|*s*) represents the probability of receiving a certain measurement *z_t_* provided that the robot is in a certain pose *s*. We have seen that in order to compute the observation model for the network of transmitters, we need to compute the model of each transmitter individually ( $\begin{array}{cc} p\left(z_{t}^{k}|s\right) & \forall \end{array}k\in \left\{1,\ldots ,n_{t}^{z}\right\}$). There are many approaches to construct observation models [1], but a common one is to express them as the similarity between the measurement received $z_{t}^{k}$ and the measurement expected to be received at each position *ẑ_s_*.

(20) $$p\left(z_{t}^{k}|s\right)=\text{sim}\left(z_{t}^{k},{\hat{z}}_{s}\right)$$

The expected measurement *ẑ_s_* can be computed using regression analysis. Regression analysis aims at estimating the relationships among variables: in this case, among the robot position and the signal power of each transmitter at that position. This relationship can be learned from training data using a wide range of regression algorithms, from which we have chosen Gaussian process (GP) regression [23].

### 5.1. Learning Observation Models Using Gaussian Process Regression

GP regression [23] has already been used with great success to build probabilistic radio maps [35,36]. Gaussian processes are a supervised learning technique; therefore, they can learn the prediction function (the map) from a training dataset. Applied to the problem of mapping the strength of a wireless signal, GP regression gives us the average and typical deviation values at every map position. Duvallet *et al.* described several of their advantages with respect to other techniques [35]. First, GPs are non-parametric, so they do not require a regression model to fit the data. Second, both linear and non-linear models may emerge from the regression (whichever fits the data best). Third, GPs are continuous, meaning that: (1) training points do not need to be discretized; (2) training points do not have to be gathered at regularly-spaced intervals; and (3) predictions can be generated for any point in the environment. Finally, contrary to other alternatives, such as *ϵ*-SVR [37], GPs correctly handle uncertainty in both the process and the estimation and naturally provide probabilistic estimations. We would like to add to this list that GPs are especially suited to solve 2D spatial regression problems, because of the use of a kernel function that can model the spatial correlation among nearby points in the environment. In addition, due to their probabilistic nature, GP regression techniques can be integrated naturally with probabilistic estimation algorithms [35,36].

As any regression technique, GP regression attempts to predict the output of a system for any arbitrary input, where outputs and inputs are continuous variables. Gaussian processes learn the prediction function from a training dataset $D=\left\{\left(d_{in}^{i},d_{\text{out}}^{i}\right)|i\in \left\{1,\ldots ,n\right\}\right\}$, which contains *n* samples of inputs $d_{in}^{i}$ and their corresponding outputs $d_{\text{out}}^{i}$. GP regression assumes that the training set is generated by a process that fulfills:

(21) $$\begin{array}{cc} d_{\text{out}}^{i}=f\left(d_{in}^{i}\right)+є, & \forall i\in \left\{1,\ldots ,n\right\} \end{array}$$

where *f* is the function that defines the system and *ϵ* is additive Gaussian noise with zero mean and variance $\sigma_{n}^{2}$.

In order to learn this function, GPs relies on a covariance function kernel $k\left(d_{in}^{p},d_{in}^{q}\right)$ that specifies the correlation among inputs. The idea behind this function is that input points that are close to each other are likely to have similar output values. There are many choices for this kernel [23], but in this paper, we have used the squared exponential kernel [35]:

(22) $$k\left(d_{in}^{p},d_{in}^{q}\right)=\sigma_{f}^{2}\exp\left(-\frac{1}{2}{\left(d_{in}^{p}-d_{in}^{q}\right)}^{t}L\left(d_{in}^{p}-d_{in}^{q}\right)\right)$$

where $\sigma_{f}^{2}$ is the signal variation and *L* is a diagonal matrix whose elements are length scale parameters that determine the strength of correlation among inputs. This kernel $k\left(d_{in}^{p},d_{in}^{q}\right)$, captured for every pair of points of the dataset, is a matrix *K*. Moreover, we will represent as k* the vector of covariances between an arbitrary input d*_in_ and the training inputs in *D_in_*.

GP regression does not compute the function f directly. Instead, it defines a distribution of probability over functions that aim at explaining the training data. For any arbitrary input point $d_{in}^{*}$, the posterior probability distribution over these functions will be [23]:

(23) $$p\left(f\left({d^{*}}_{in}\right)|{d^{*}}_{in},D\right)∼N\left(\mu^{*},\sigma^{*2}\right)$$

(24) $$\mu^{*}=k^{*t}{\left(K+\sigma_{n}^{2}I\right)}^{-1}d_{\text{out}}$$

(25) $$\sigma^{*2}=k\left({d^{*}}_{in},{d^{*}}_{in}\right)-k^{*t}{\left(K+\sigma_{n}^{2}I\right)}^{-1}k^{*}$$

That is, for any input $d_{in}^{*}$, GP regression predicts a normal distribution centered in μ* (most probable output), with a typical deviation of σ* (that models both the data noise and the uncertainty of the prediction). All of the parameters of the GP regression can be learned from training data by maximizing the log marginal likelihood of the observations conditioned on the parameters [23].

**Figure 4:**
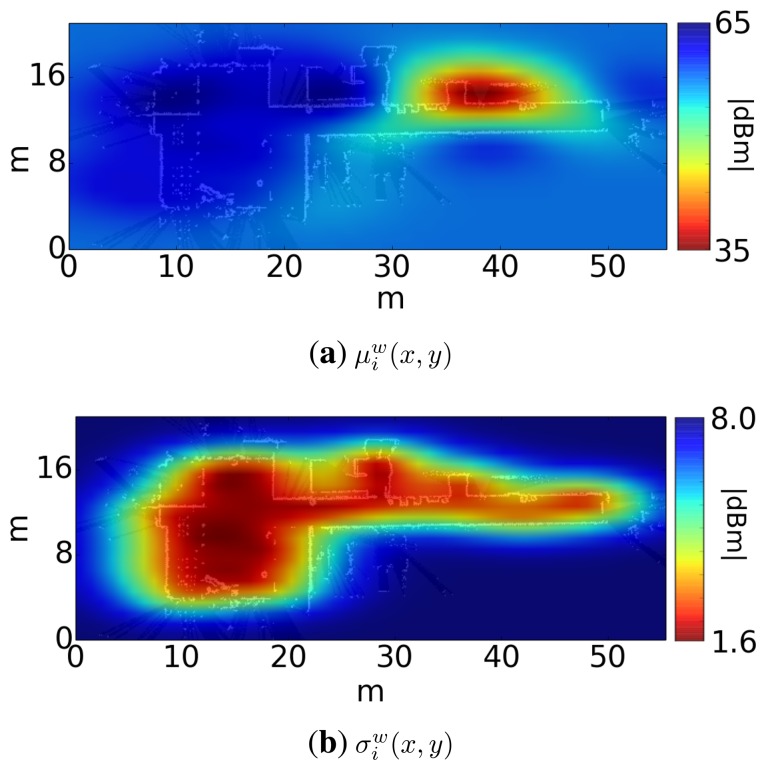
Output of the Gaussian process regression for a sample mote. (**a**) The lowest values (red) correspond to the highest power. It is clear that the APwas located near the position (40 m,16 m); (**b**) The lowest values (red) correspond to the lowest typical deviation power.

In our case, we will have a training dataset for each transmitter *k*. This training dataset will be $D^{k}=\left\{\begin{array}{cc} \left(x_{t},y_{t}\right); & z_{t}^{k} \end{array}\right\}$. Each sample of the training set consists of an output $z_{t}^{k}$ (power of the *k*-th AP received from the scan at time *t*), associated with an input (*x_t_*, *y_t_*) (position where the scan took place). With this training set, the regression computes for each transmitter the functions *μ^k^*(*x*, *y*) and *σ^k^*(*x*, *y*), which represent the average and the typical deviation of the signal strength of the *k*-th transmitter across the environment. Figure 4 shows a representation of these functions for a sample transmitter.

Provided these functions, the observation model that we will use for each transmitter is:

(26) $$p\left(z_{t}^{k}|s\right)\propto \frac{1}{\lambda \sigma^{k}\left(x,y\right)\sqrt{2\pi }}\exp\;\left[-\frac{1}{2}{\left(\frac{z_{t}^{k}-\mu^{k}\left(x,y\right)}{\lambda \sigma^{k}\left(x,y\right)}\right)}^{2}\right]$$

where λ is a parameter that scales the typical deviation estimated by the GP regression. Therefore, it modifies the confidence that we have in the sensor model (the greater λ, the lower the confidence and the higher the tolerance towards noise). With this in mind, the observation model when using all of the transmitters (Equation (10)) becomes:

(27) $$p\left(z_{t}|s\right)\propto \sum_{k=1}^{n_{t}^{z}}\frac{1}{\lambda \sigma^{k}\left(x,y\right)\sqrt{2\pi }}\exp\;\left[-\frac{1}{2}{\left(\frac{z_{t}^{k}-\mu^{k}\left(x,y\right)}{\lambda \sigma^{k}\left(x,y\right)}\right)}^{2}\right]$$

**Figure 5:**
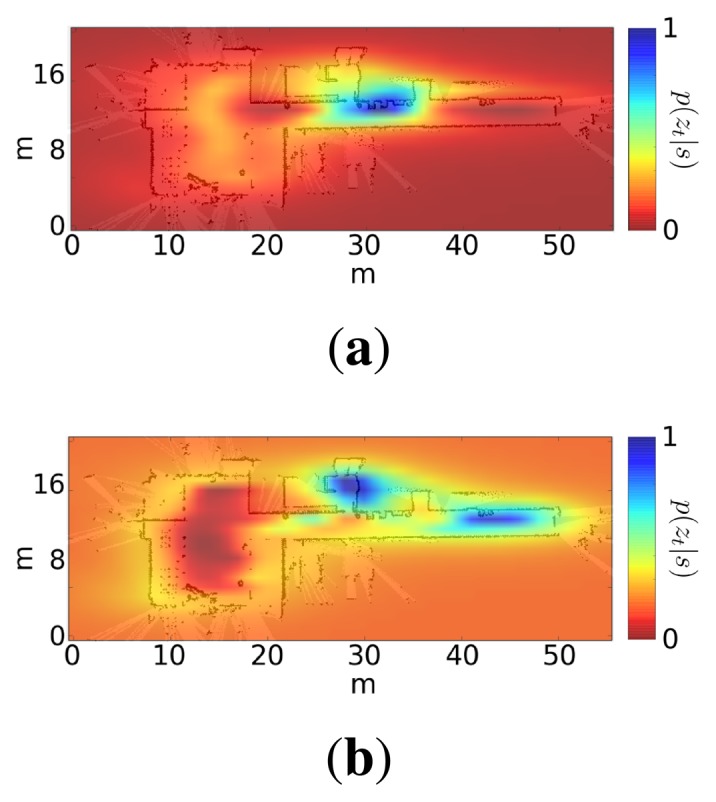
Likelihood distribution provided by the wireless observation model at two different positions (highest values in blue; lowest values in red).

Figure 5 shows two examples of this likelihood distribution when the robot is at two different positions. Note that in both cases, the distribution is multi-modal, but there is usually an area that concentrates most of the likelihood.

### 5.2. Collection of Calibration Data and Training of Observation Models in Practice

**Figure 6:**
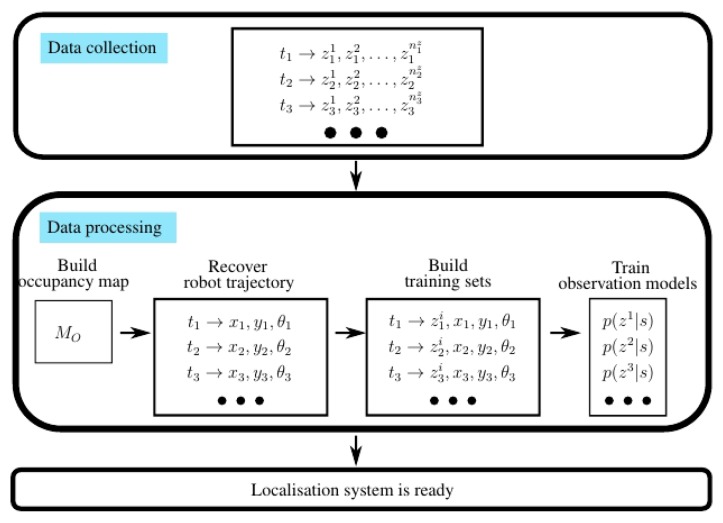
Observation model calibration procedure.

**Figure 7:**
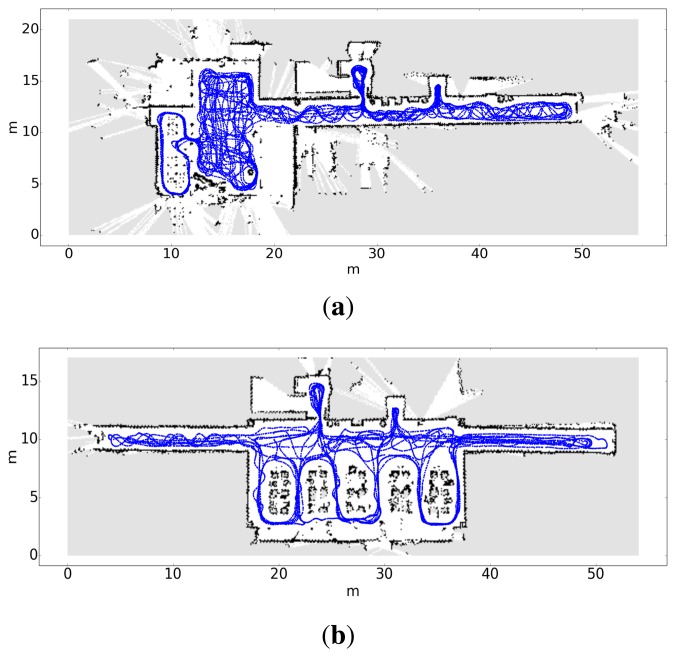
Trajectory followed by the robot during the capture of the experimental data. (**a**) Trajectory followed in the first floor; (**b**) Trajectory followed in the second floor.

In order to train the observation models, we must: (1) capture a number of measurements at different points of the environment (calibration data); and (2) build the training sets. These last steps require us to relate each measurement with the position where it was measured. There exists a number of alternatives to accomplish this. For instance, there exist systems that provide the position of the robot at every instant (e.g., external infrared cameras for 3D marker tracking, such as Tracking Tools from Natural Point). However, these systems are extremely expensive and adequate only for small spaces. As an alternative, a user may indicate where each measurement took place, but this method has some serious drawbacks: it is tedious, time consuming and error prone. In order to overcome these issues, we follow the procedure depicted in Figure 6:

1. Collect the calibration data by moving the robot around the environment. This data includes: laser data, odometry data and signal power data captured by the wireless receiver. The user may move the robot either: (1) with a joystick or; (2) with our person-following behavior [38] with gesture-based interaction and voice feedback [39]. We have seen that non-expert users are able to perform this step successfully using either of each method.
2. Process the collected data off-line.
  (a) Build a map of the environment. This map will only be used as a frame of reference for the calibration data and for the estimations of the localization algorithm. To construct this map, we use an SLAM algorithm (simultaneous localization and mapping) with laser and odometry information.
  (b) Compute the trajectory followed by the robot and associate each pose with its timestamp (*x_t_*, *y_t_*, *θ_t_*). Figure 7 contains two examples of maps and the trajectory followed by the robot during the calibration stage.
  (c) Build the training sets. Each training set contains the signal power received from each transmitter, associated with the pose where the data was captured.
  (d) Train the observation models.

## 6. Experimental Results

The purpose of the experiments is to compare WiFi localization with localization with motes. We will perform two different experiments:

1. Analysis of the performance of localization with motes and WiFi localization using a fixed transmission power in both cases: We want to compare their performance to see whether localization with motes could be used in the context of mobile robot localization. It is known that the performance of WiFi localization depends on the quality of the WiFi card placed on board the robot [13]. Therefore, to obtain a more reliable result, we will perform this experiment by using three different WiFi cards.
2. Analysis of the performance of the localization with motes when using different transmission powers: We will also explore if using motes that change their transmission power dynamically increases the performance of the localization algorithm. This is important, because if this is the case, robots could change the transmission power of motes to achieve a better performance (e.g., active localization, where the robot may ask the motes to modify the transmission power in order to validate or reject localization hypotheses).

Both experiments will be performed using the same dataset, obtained by the process that we will describe in the following section.

### 6.1. Setup and Methodology

We have performed experiments on two different floors of the CITIUS research center (Centro Singular de Investigación en Tecnoloxías da Información da Universidade de Santiago de Compostela, Spain). We have deployed 6 WiFi access points and 6 motes on each floor (each mote was placed near an AP, to achieve a fair comparison). The WiFi APs are TP-Link TD-W8968 with EIRP < 20 dBm (equivalent isotropically-radiated power). These APs have 2 dipole antennas of 5 dBi each, while each mote has a 1.8-dBi monopole antenna (Section 3). The radiation pattern of each WiFi antenna in the azimuth plane (the one relevant in our system) is similar to the pattern of each mote antenna (Figure 1). On the other hand, we have used a Pioneer P3DX robot equipped with a SICK-LMS100 laser, 1 mote (receiver) and 3 different commercial WiFi receiver cards.

To collect the experimental data, we have moved the robot around the environment with a joystick. During this process, the robot recorded the information received from the odometry encoders, the laser and the signal power received by the mote and the WiFi cards. Table 1 shows a summary of the data collected. Note that one of our experiments involve changing the power transmission of the motes. In principle, we would have to repeat the data capture stage as many times as the number of transmission powers that we would like to explore (re-configuring the motes every time). Instead, we have programmed the transmission motes to send a new package of data each 250 ms. Each package is transmitted with a different power: −40 dBm, −30 dBm, −20 dBm, −10 dBm, 0 dBm, 5 dBm, 7 dBm and 10 dBm. Therefore, the first package is transmitted at −40 dBm; after 250 ms, a new package is transmitted at −30 dBm, and so on. The cycle is repeated after 2 s, after the package at 10 dBm is transmitted. On the other hand, the robot's mote receives this packages, and every 2 s, it sends to the robot a vector containing the signal power of the last packages received. This way, to analyze the performance under a certain transmission power, we just have to discard all packages except those transmitted at the power that we want to analyze. Moreover, these same data can be used to analyze the performance when the transmission power changes periodically.

After the experimental data were captured, we divided them into two subsets:

- Training set: This represents approximately the first 33% of the dataset. We used this set to construct the map (with the laser and odometry information) and to calibrate the observation models of the mote and the 3 WiFi cards (with the signal power received by each of them).
- Testing sets: The remainder of the dataset was divided randomly into 20 parts of 120 s each. Each part was used to test the performance of our localization algorithm.

In each experiment, we execute the algorithm using each of the test sets (20 times each, to carry out statistical analyses). In each execution, the robot starts with no knowledge about its pose (global localization), until the algorithm converges to a pose estimate (tracking). The localization algorithm estimates the trajectory followed by the robot using the robot odometry and the signal power received from the WiFi APs or motes (depending on the case). Finally, we compare this trajectory with the real trajectory (ground-truth), in order to evaluate:

1. Error in position and orientation (*e_xy_* and *e*_θ_): the difference between the pose estimated by the algorithm and the ground-truth (the lower the better).
2. Convergence ratio (%*t_loc_*): the percentage of time that the algorithm provides an estimation of the pose (the higher the better). We consider that the algorithm has converged when the clustering step discovers a sufficiently important cluster of particles (Section 4.3).

We have always used the following parameters: *n^p^* = 2000 particles, *α^s^* = 0.01, *α^f^* = 0.1, *dist*_*th* − *xy*_ = 5 *m*, *dist*_*th* − *θ*_ = *π*/2, Ω_*th*_ = 3/4. We have executed our algorithm with a period of 333 ms (control cycle), enough to ensure the correct performance of the tasks carried out by our robot (e.g., planning and navigation). The execution of the localization algorithm takes approximately 15 ms of the control cycle. This indicates that our algorithm can work in real time.

### 6.2. Ground-Truth Collection

We need to know the trajectory followed by the robot in each test set. This trajectory is called the ground-truth. In order to collect it, we have followed a procedure inspired in other works [40]. First of all, an expert initializes the localization algorithm with the initial true pose of the robot during the trajectory, which is known. Then, the algorithm processes the laser and odometry information and generates the trajectory followed by the robot. Finally, each pose of the trajectory is either accepted or rejected by the expert, who compares the real laser signature with the signature expected from that pose (visual inspection).

We have evaluated the accuracy of the ground-truth obtained by this procedure. We have moved the robot around the environment, forcing its trajectory to pass over 8 checkpoints. Then, we measured the real robot pose at each checkpoint. Finally, we have compared each real pose with the corresponding pose estimated by the ground-truth construction procedure. We have obtained a median difference of 0.254 m in position and 0.033 rad in orientation. We are aware that the use of a high precision localization system would have been a better option to build the ground-truth. However, we did not have access to a system like this that is able to work in such big areas. Moreover, we believe that the results obtained are adequate for the purposes of this paper.

### 6.3. Results: Performance of Motes vs. WiFi at a Fixed Transmission Power

**Figure 8:**
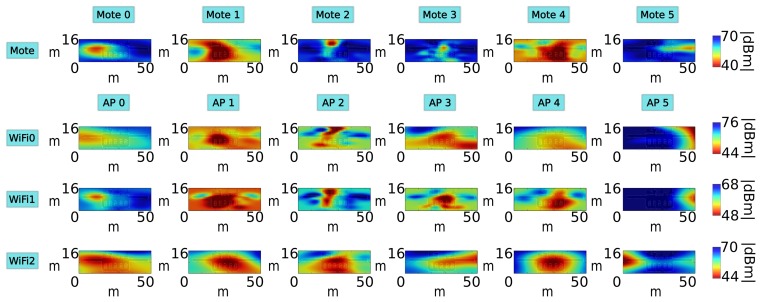
Radio maps generated with one of the datasets. Each row represents a different receiver (mote or WiFi card) and each column a different transmitter (mote or WiFi AP).

In this experiment, we have analyzed the performance of the localization algorithm using motes and WiFi APs at a fixed transmission power (we have used the same power for motes and WiFi APs). As we have explained, the performance of WiFi localization depends on the quality of the WiFi card placed on board the robot, so we will use three different WiFi cards in this experiment. Figure 8 shows the radio maps of one floor, for each receiver. We can see that the radio maps depend greatly on the hardware of the receiver [13]: each radio map is different from the rest, not only between motes and WiFi, but among the three WiFi receivers, as well.

**Figure 9:**
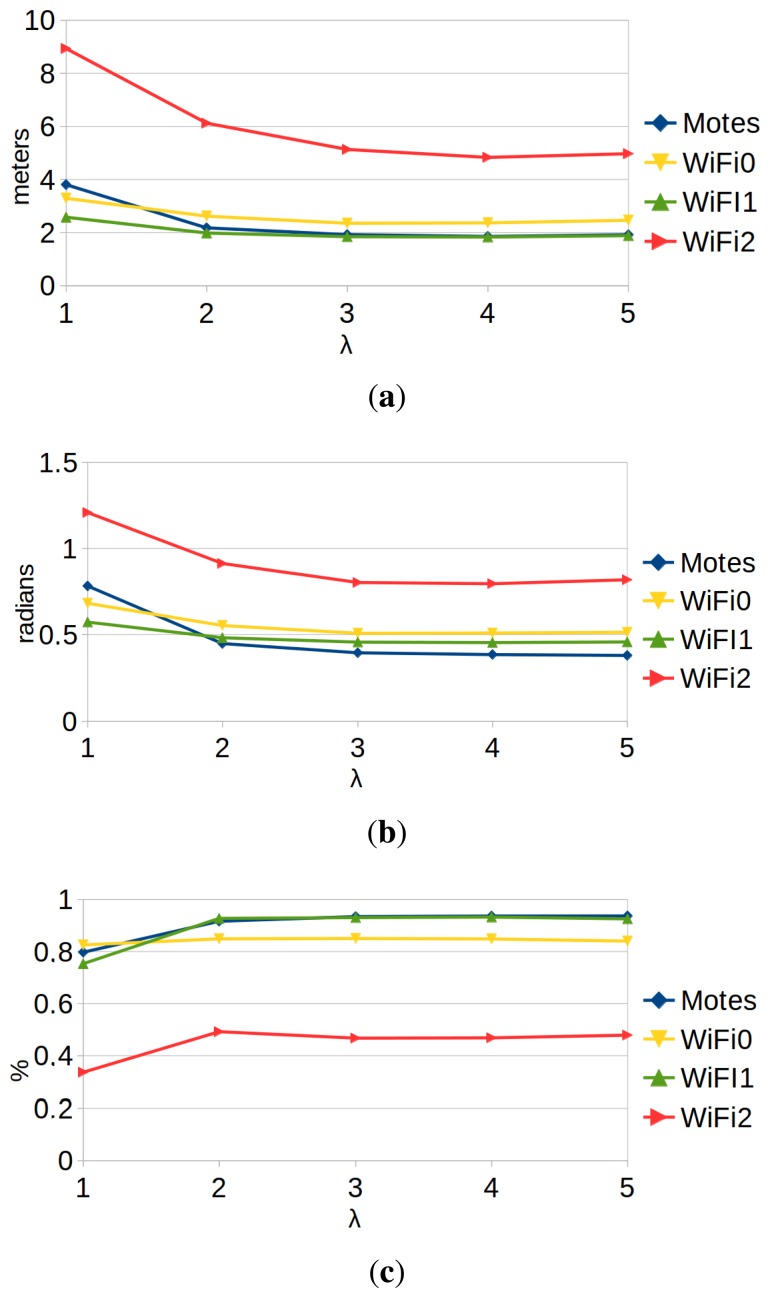
Comparison of the performance of the algorithm using the motes (blue) and each WiFi card (yellow, green, red). (**a**) *e_xy_;* (**b**) *e_θ_*; (**c**) *%t_loc_*.

Figure 9 shows the performance results of the localization with motes and WiFi. The experiments were performed using different values of the parameter λ. This parameter scales the typical deviation estimated by the GP regression (Equation (27)). Therefore, it modifies the confidence that we have in the sensor model (the greater the λ, the lower the confidence and the higher the tolerance towards noise). We can draw the following conclusions:

1. Higher values of λ tend to give better results. On the one hand, this happens because particle filters work better when they are conservative about the confidence in the observation models [1]. On the other hand, the training data were captured during a limited amount of time under very stable circumstances. Therefore, any regression algorithm will tend to over-fit, but the λ parameter helps to mitigate this problem. However, the value of the parameter cannot be arbitrarily large: the larger λ, the less information the observation model provides. Results show that the best trade-off is a value of λ between three and four.
2. WiFi localization results depend greatly on the hardware that we use: we have obtained very different results with each WiFi receiver.
3. Performance results of localization with motes are at least as good as the best results of WiFi localization. Therefore, our proposal is just as valid as WiFi localization to be used for wireless robot localization.

### 6.4. Results: Varying the Transmission Power

In this experiment, we have compared the performance of the localization algorithm using: (1) motes with different transmission powers; (2) motes that change the transmission power periodically; and (3) the best WiFi receptor of the previous experiments, whose transmission power cannot be modified. In this case, we could only use four out of the six total transmitter motes, because the other two did not allow us to change the transmission power due to firmware restrictions. Obviously, we used only the four WiFi APs that were near the selected motes, to ensure a fair comparison.

Note that we did not have to repeat the data capture stage, because the motes were already sending a new package of data each 250 ms, each package with a different power (−40 dBm, −30 dBm, −20 dBm, − 10 dBm, 0 dBm, 5 dBm, 7 dBm and 10 dBm). Therefore, to analyze the results with a certain fixed transmission power, we just have to discard all of the packages, except those transmitted with that power. Similarly, to analyze the results with a varying transmission power, we can consider that we have eight ‘virtual’ motes for every real mote (because each real mote transmits at eight different powers per cycle). Therefore, to analyze the performance when varying the transmission power, we have trained a total of 32 ‘virtual’ observation models, and we have just integrated each mote package using the observation model of its corresponding ‘virtual’ mote.

**Figure 10:**
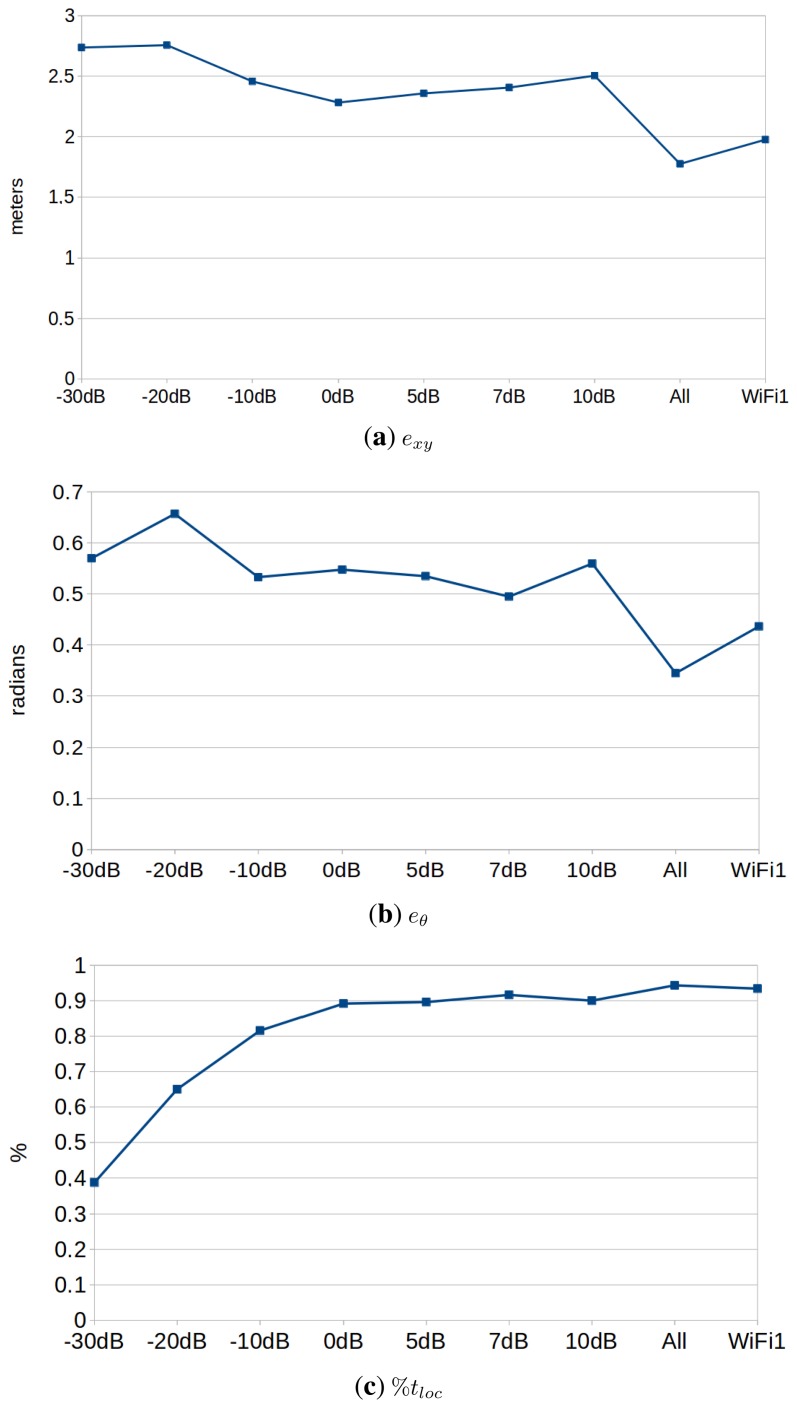
Comparison of the performance of the algorithm using: (**a**) motes with different fixed transmission powers (tagged as −30 dBm, −20 dBm, −10 dBm, 0 dBm, 5 dBm, 7 dBm or 10 dBm); (**b**) motes that change their transmission power periodically (tagged as ‘all’); and (**c**) the WiFi card that performed better in the previous experiment (tagged as WiFi1). We have discarded the results obtained when using a transmission power of −40 dBm, because the algorithm did not converge most of the times.

Figure 10 shows the performance results in this experiment, and Table 2 ranks the performance of each configuration. The experiments were performed using a value of λ = 4. We can draw the following conclusions:

1. The best configuration is always the one that uses varying transmission power, and the difference is significant when considering *e_xy_* and *e_θ_*. This suggests that the use of varying transmission powers provides useful information that improves the localization results. This opens the door for future improvements in the line of active localization. Under this paradigm, the robot would be able to modify the transmission power of the motes in order to discard localization hypotheses proactively.
2. The second best configuration is, in this case, the one obtained when using the WiFi card. This contradicts partially the results obtained in the previous section, where the motes performed just as well as the best WiFi card. Anyway, just as in the previous case, the results may vary depending on a number of factors: the number of APs, the distribution in the environment, environmental conditions, *etc*.
3. There is no significant difference among the different transmission powers analyzed, considering *e_xy_* and *e_θ_*. However, powers above 0 dBm perform better on the convergence rate results (%*t_loc_*). This makes sense, because motes with low transmission power can only be received in the surroundings of the mote. Therefore, the algorithm will only converge when passing near one of them. We can extract two recommendations from these results. First of all, motes should use a transmission power of 0 dBm, which is the lowest one with the best results. Second, lower transmission powers might be used, as well (e.g., proximity-based localization), but a greater number of motes should be used.

## 7. Conclusions and Future Work

In this paper, we have demonstrated that the use of transmitters with varying transmission powers can improve the performance of wireless positioning systems. We have proven this result with a wireless localization system for mobile robots. In particular, we have designed a system based on motes that operate in the 433-MHz band. The motes allow one to modify several transmission parameters and have a very small power consumption. We have presented the hardware and software design of the motes, and we have described how to integrate them with a localization algorithm based on particle filters.

The experimental results described in this paper show that the localization with motes is at least as good as WiFi localization. This shows that mote localization is a viable alternative to WiFi localization. Nevertheless, working with motes provides some interesting characteristics that make them very appealing: (1) control over the software and hardware of the mote; (2) homogeneous hardware; (3) full control of the communication capabilities; (4) low power consumption, *etc*. However, our objective is not to substitute WiFi localization systems; therefore, we believe that both technologies could even co-exist in a robotics application, in order to increase the redundancy of the system.

In addition, experimental results have shown that different transmission powers result in different performances. Particularly, the performance was optimum when using transmission powers above 0 dBm. Most importantly, we have analyzed the performance of the algorithm when using motes that vary their transmission power periodically. The results show that this configuration improves the results significantly with respect to any other configuration. This suggests that the use of varying transmission powers provides useful information that improves the localization results. This result is as valid for our motes as it is for other wireless technologies, provided that they allow the modification of the transmission power. This opens the door to explore new research lines, such as active localization, where the robot can proactively modify the power of the transmitters, in order to discard localization hypothesis actively.

In the future, we will use the mote system to provide communications among our robots and other intelligent elements, such as a network of intelligent cameras that we have developed [43,44]. In addition, we will further explore the use of patterns of varying transmission powers to improve the robot localization. Finally, we will explore the use of the motes in active localization strategies, where the robot will ask the motes to vary their power to improve the localization results.

## Figures and Tables

**Table 1:** Summary of each experimental trajectory: length (in seconds and meters) and the number of readings of the odometry, laser and WiFi.

**Experiment**	**Length**	**Odometry**	**Laser**	**Motes**	**WiFi0**	**WiFi1**	**WiFi2**
1	2330 s/878 m	23,245	115,846	1150	1673	1691	1155
2	2894 s/1245 m	28,898	144,136	2756	2093	2108	1416

**Table 2:** Ranking of the second experiment according to different statistical measurements using the Friedman aligned ranks method [41] (*p*-value = 0.05). We highlight in boldface the winning configuration. We separate configurations with horizontal rows when there is a significant different among them (*post hoc* Holm test [41] with *α* = 0.05). These statistical tests were performed using the STAC web tool [42]. We have analyzed the performance of the algorithm using the motes with different transmission powers (tagged as −20 dBm, −10 dBm, 0 dBm, 5 dBm, 7 dBm and 10 dBm), with varying transmission power (tagged as ‘all’) and with the best WiFi card of the previous experiment (tagged as WiFi1). We have discarded the results obtained when using a transmission power of −40 dBm (because the algorithm did not converge most of the times) and −30 dBm (because the algorithm did not converge sometimes).

**Ranking**	*e_xy_*	*e_θ_*	%*tloc*
1 (best)	**All**	**All**	**All**

2	WiFi1	WiFi1	**WiFi1**

3	0 dB	7 dB	**7 dB**
4	5 dB	−10 dB	**10 dB**
5	7 dB	10 dB	**0 dB**
6	−10 dB	5 dB	**5 dB**

7	10 dB	0 dB	−10 dB
8 (worst)	−20 dB	−20 dB	−20 dB
